# Development of a Three-Dimensional Carotid Ultrasound Image Segmentation Workflow for Improved Efficiency, Reproducibility and Accuracy in Measuring Vessel Wall and Plaque Volume and Thickness

**DOI:** 10.3390/bioengineering10101217

**Published:** 2023-10-18

**Authors:** Yuan Zhao, Mingjie Jiang, Wai Sum Chan, Bernard Chiu

**Affiliations:** 1 Department of Electrical Engineering, City University of Hong Kong, Hong Kong; yuazhao2-c@my.cityu.edu.hk (Y.Z.); minjiang5-c@my.cityu.edu.hk (M.J.); wschan484-c@my.cityu.edu.hk (W.S.C.); 2 Department of Physics & Computer Science, Wilfrid Laurier University, Waterloo, ON N2L 3C5, Canada

**Keywords:** carotid atherosclerosis, 3D ultrasound (3DUS), vessel wall volume (VWV), vessel-wall-plus-plaque thickness (VWT), intra-observer reproducibility, patient-based partition, time-based partition

## Abstract

Automated segmentation of carotid lumen-intima boundary (LIB) and media-adventitia boundary (MAB) by deep convolutional neural networks (CNN) from three-dimensional ultrasound (3DUS) images has made assessment and monitoring of carotid atherosclerosis more efficient than manual segmentation. However, training of CNN still requires manual segmentation of LIB and MAB. Therefore, there is a need to improve the efficiency of manual segmentation and develop strategies to improve segmentation accuracy by the CNN for serial monitoring of carotid atherosclerosis. One strategy to reduce segmentation time is to increase the interslice distance (ISD) between segmented axial slices of a 3DUS image while maintaining the segmentation reliability. We, for the first time, investigated the effect of ISD on the reproducibility of MAB and LIB segmentations. The intra-observer reproducibility of LIB and MAB segmentations at ISDs of 1 mm and 2 mm was not statistically significantly different, whereas the reproducibility at ISD = 3 mm was statistically lower. Therefore, we conclude that segmentation with an ISD of 2 mm provides sufficient reliability for CNN training. We further proposed training the CNN by the baseline images of the entire cohort of patients for automatic segmentation of the follow-up images acquired for the same cohort. We validated that segmentation with this time-based partitioning approach is more accurate than that produced by patient-based partitioning, especially at the carotid bifurcation. This study forms the basis for an efficient, reproducible, and accurate 3DUS workflow for serial monitoring of carotid atherosclerosis useful in risk stratification of cardiovascular events and in evaluating the efficacy of new treatments.

## 1. Introduction

Stroke is one of the leading causes of both death and disability worldwide, resulting in 6.55 million deaths and 143 million disability-adjusted life-years (DALYs) in 2019 [1]. With the aging of the population and the epidemiological transition from infectious to non-communicable diseases in low-income and middle-income countries, the global burden of stroke and subsequent post-stroke care will significantly increase in the future [2]. In addition to the serious social, emotional, and financial repercussions on the individuals who suffer stroke and their families, stroke also has a substantial impact on health and social care services. The direct and indirect cost of stroke in the USA was estimated to be $103.5 billion per year [3]. Most strokes are ischemic [4], and the main culprit is the blockage of the cerebral artery by an embolus. Carotid atherosclerosis is a chronic inflammatory disease involving the gradual accumulation of plaque within the inner wall of the carotid arteries. The disease is characterized by endothelial dysfunction, mononuclear accumulation, smooth muscle cell and fibrous matrix proliferation, leading to the formation of atherosclerotic plaques [5], which is the major source of emboli in the form of platelet aggregates and plaque debris. Interventions with dietary and medical treatments are effective in preventing most vascular events associated with carotid atherosclerosis [6,7,8]. Therefore, sensitive and cost-effective assessment tools or biomarkers for carotid atherosclerosis are essential for measuring carotid atherosclerosis burden, managing patients exposed to high risk of vascular events, and validating treatment strategies in clinical trials.

Ultrasound imaging technique has been used for quantitative assessment of carotid atherosclerosis burden. Carotid intima-media thickness (IMT) measured from the longitudinal view of two-dimensional ultrasound (2DUS) images has been used for more than 30 years. However, IMT is a weak predictor of cardiovascular events [9], and the annual change of IMT (∼0.015 mm) is too small to be detected within a clinically affordable time frame [10]. In addition, IMT measurements assess the intima-media thickening measured in the common carotid artery (CCA), which is more often caused by hypertension-induced medial hypertrophy and therefore does not directly indicate atherosclerosis [11]. Total plaque area (TPA) directly quantifies plaque burden from 2DUS images and is more sensitive than IMT in predicting stroke [12]. However, both IMT and TPA are obtained from 2DUS examinations that require an operator to locate a 2D imaging plane to be scanned. The operator variability in selecting the imaging plane makes 2DUS suboptimal for serial monitoring of atherosclerosis.

Mechanical three-dimensional ultrasound (3DUS) imaging technique has been developed that uses a motorized mechanical device to translate the transducer along a patient’s neck, capturing a series of successive 2D images, which are then reconstructed into 3D images [13]. The availability of 3DUS obviates the need for the clinician to mentally transform multiple 2D ultrasound images to form a 3D impression of the complex structure of the carotid vessel and plaque and allows for objective and quantitative 3D volume measurements to be made. These measurements include total plaque volume (TPV) and vessel wall volume (VWV), which were shown to be more sensitive to changes in carotid atherosclerosis than IMT and TPA [9,14], as the thickness, length and circumferential extent of plaque burden can be measured by 3DUS. TPV and VWV were shown to be sensitive to medical treatment effect and reduce the sample size and duration required to establish the treatment effectiveness [15,16,17], thereby making clinical trials more cost-effective and shortening the time required to withhold effective treatments from patients who need them. The progression of TPV was shown to predict cardiovascular events in a study involving over 300 patients, whereas TPA progression did not prevent events [9], providing evidence to support the increased sensitivity of TPV in risk stratification. VWV shows higher inter-scan reproducibility than TPV as media-adventitia boundaries (MAB) and lumen-intima boundaries (LIB) are easier to be segmented from 3DUS than plaque [18], and can be used to assess population without measurable plaques [19]. The vessel-wall-plus-plaque thickness (VWT) is the point-by-point measurement of the distance between points on the MAB and the corresponding points on the LIB; it can be mapped onto a 2D L-shaped carotid map to show the spatial distribution of the vessel wall and plaque changes over the carotid artery and for quantitative analysis [20,21]. VWT-based metrics were demonstrated to be more sensitive than VWV and TPV to the effect of dietary intervention [22,23].

The pre-requisite for VWT and VWV measurements is the availability of LIB and MAB. Although they can be segmented from 3DUS images manually and manual segmentation has been performed for previous clinical studies [14,16,18], manual segmentation is prohibitively time-consuming even for small-scale clinical studies involving tens of patients. Observers trained in our group take five hours to segment the MAB and LIB in a 3DUS volume with a 1 mm interslice distance (ISD) for a coverage of 25 mm (15 mm of CCA and 10 mm of ICA). The small-scale study involving 56 patients in a placebo-controlled study of the effect of Vitamin B supplement [22] requires the segmentation of 224 volumes (4 volumes per patient: left and right carotids at baseline and follow-up image sessions) and would take more than a thousand hours of manual segmentation time. In addition, the reliability of manual segmentation depends heavily on the expertise of individual observers. For this reason, observers are required to be trained for the ability to provide reproducible measurements for VWV [24] and VWT [23] for the same set of images on different segmentation sessions, which are separated for a period to minimize the effect of memory. These trainings typically take weeks to complete. Without sufficient training on manual segmentation, the observer would not be able to produce the reliable segmentation required to train a segmentation CNN. The need for more efficient ways to delineate LIB and MAB reproducibly motivates the development of semi-automatic or automatic segmentation algorithms designed to minimize human intervention and improve observer reproducibility while substantially reducing the segmentation time required.

Early approaches are based on deformable models that require extensive interaction (2–14 min) to initialize the models [25,26,27]. These methods have been phased out by recently developed deep learning algorithms that require fewer human interactions to generate segmentation results. Menchón-Lara et al. [28] used multi-layer perceptrons trained under the scaled conjugate gradient algorithm to delineate MAB and LIB of the common carotid arteries (CCA) from 2D longitudinal carotid US images. Zhou et al. [29] proposed a semiautomatic CCA segmentation method using the dynamic convolutional neural network and U-Net to segment MAB and LIB, respectively, from 3D carotid US images. The same group [30] proposed another segmentation network combining a 3D deep convolutional neural network and a continuous max-flow module to segment CCA from a manually identified region of interest (ROI). Lin et al. [31] used the cross-shaped window (CSWin) transformer to optimize a standard U-shaped neural network and proposed a U-CSWT model to segment the LIB and MAB of CCA in 3DUS images. However, the above deep learning approaches made no effort in segmenting the internal carotid arteries (ICA), thereby precluding vessel wall quantification at the ICA. VWV and VWT measured only at CCA may be less sensitive to treatment effect than those measured in previous studies that involved both CCA and ICA [16,22], with carotid plaques typically more prevalent at ICA [32]. Jiang et al. [33] previously developed a two-channel U-Net, driven by a novel loss function called the adaptive triple Dice loss (ATDL) function that can segment LIB and MAB simultaneously. The segmentation performed on CCA slices was fully automatic, whereas the ICA was enclosed by a manually identified bounding box that ensured the ICA was segmented, instead of the external carotid artery (ECA), which are less relevant to cardiovascular events [34] and typically not measured for atherosclerosis [9,22,35]. As the application of this algorithm allows automated quantification of VWV and VWT at the CCA and ICA, the segmentation strategies proposed in the current study were built upon this algorithm.

The goal of the current study is to evaluate and optimize the efficiency, reproducibility, and accuracy of the 3DUS carotid segmentation and quantification workflow. The major bottleneck in the efficiency of the deep-learning framework is the requirement to train the network by manual segmentation of MAB and LIB. One strategy to reduce the manual segmentation time is to increase the inter-slice distance (ISD) between adjacent segmented boundaries (so that fewer axial images are needed to be segmented), but doing so may reduce the reproducibility of carotid measurements. Although the effect of ISD on mean plaque volume has been investigated [36], the current study is the first to investigate the effect of different ISD settings on the reproducibility of MAB and LIB segmentations and the associated VWV and VWT measurements.

Secondly, images used to train deep networks are typically randomly partitioned in the training, validation, and testing sets. An alternative way to partition data for studies involving serial monitoring of patients that may lead to higher segmentation performance would be to train the network on baseline images of patients with manual segmentation and test it on follow-up images. The network trained by baseline images had been exposed to the geometry of the carotid arteries in the entire cohort. While segmenting the follow-up images of the same cohort, we hypothesize that the segmentation accuracy of the network would be higher than a network trained by a randomly selected training set as the network had seen the images of the same set of patients, although at a different time point. This hypothesis, if validated, would suggest a clinically feasible workflow in which the network is trained by manually segmented baseline images for automatic segmentation of the follow-up images. This new workflow would be even more advantageous in serial monitoring of atherosclerosis development in which multiple images of the same patient acquired over time are assessed, as the model trained at baseline can be applied in multiple follow-up imaging sessions. In this study, we compared the segmentation performance of the time-based partition strategy, in which manually segmented boundaries are provided for baseline volumes of all patients investigated for network training, with the patient-based partition strategy, in which manually segmented boundaries are provided for the baseline and follow-up volumes of half of the patient population for training. Specifically, we compared the segmentation accuracy of the time-based and patient-based partition strategies and the VWV and VWT measured from the boundaries segmented by these two partitioning approaches. Quantified assessment of VWT and VWT measurements generated by the two partitioning strategies were evaluated by comparison with the corresponding measurements obtained from manual segmentation. VWV and VWT measured from manual segmentation were shown to be sensitive to medical [16,21] and dietary interventions [22,23]; if comparable VWV and VWT measurements can be made with the proposed automated workflow, it would give rise to more efficient analysis of the progression/regression of carotid atherosclerosis in clinical studies involving treatment effect monitoring and evaluation.

The remaining sections of the paper are organized as follows. Section 2 is the method section that includes a description of the data set used in this study, the methodology with which we evaluated manual segmentation variability, a description of the segmentation algorithm, the conventional and the proposed data partitioning strategies, and the statistical analyses involved. The results of the study are presented in Section 3. A discussion and the conclusion of the study are provided in Section 4 and Section 5, respectively.

## 2. Methods

### 2.1. Study Population and Image Acquisition

The patients being followed up in the Stroke Prevention Clinic or the Premature Atherosclerosis Clinic at the University Hospital (London, ON, Canada) were recruited by Stroke Prevention & Atherosclerosis Research Center at Western University (London, ON, Canada) and voluntarily provided written informed consent to the research protocol approved by the local Human Research Ethics Board (approval number 12401E). These subjects had a history of risk factors for adverse vascular events, such as hypertension or hyperlipidemia, or a history of vascular events, such as stroke or transient ischemic attack. Two-dimensional ultrasound scans were acquired from the clavicle to the mandibular angle to measure the total plaque area of the carotid artery and patients with a baseline total plaque area between 40 and 600 mm${}^{2}$ (the first tertile in the patient population) [9] were involved in this study.

Participants attended the research clinic for 3DUS scans at baseline and one year later. A total of 84 3DUS volumes with a mean voxel size of 0.21×0.21×0.35 mm acquired at baseline and a follow-up session was used in this study. The high-resolution 3D ultrasound images were collected by an ATL HDI 5000 medical ultrasound machine (Philips, Bothell, WA, USA) equipped with an L12-5 transducer (Philips, Bothell, WA, USA). The transducer was mounted on a linear motor and moved about 4.0 cm vertically along the subject’s neck at a uniform speed in about 8 s. [13]. The 2D ultrasound images centered around the bifurcation were digitized from video frames and then reconstructed into 3DUS images [37].

### 2.2. Manual Segmentation

**Figure 1:**
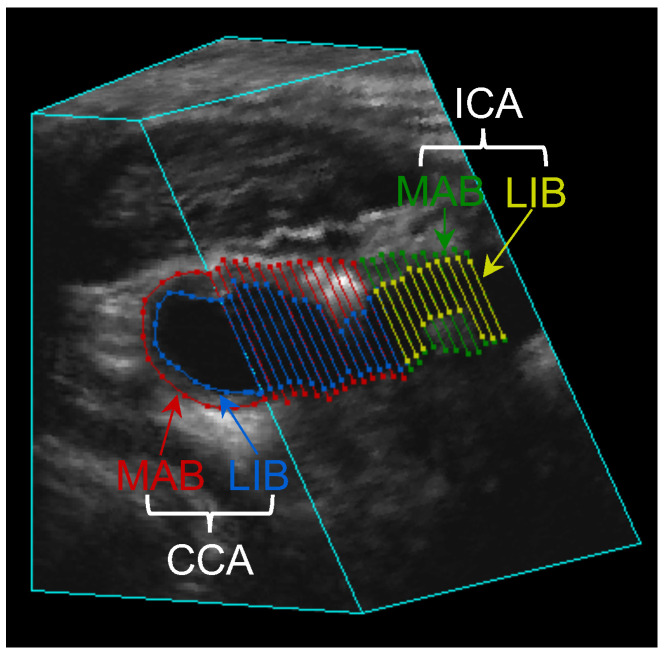
The 3D ultrasound image with MAB and LIB of the CCA and ICA segmented at the 1 mm ISD setting.

The manual segmentation of the 3DUS images was performed according to the workflow described in Krasinski et al. [16]. Briefly, the trained observer reviewed the 3DUS volume from different angles to understand the overall geometry of the carotid artery and the distribution of carotid plaques. The carotid bifurcation was marked as a reference point. The CCA and ICA were then segmented on axial image slices of the 3DUS volume, which is approximately perpendicular to the longitudinal axis of the CCA, with a fixed inter-slice distance (ISD). We varied the ISD and studied the effect of different ISDs on segmentation and measurement reproducibility. Segmentation of the LIB and MAB started at the bifurcation, extending proximally for 15 mm from the bifurcation to cover the CCA and 10 mm distally from the bifurcation to cover the ICA. Figure 1 shows an example of a 3D ultrasound image in which the MAB and LIB of the CCA and ICA have been manually segmented with an ISD of 1 mm.

We investigated the segmentation and measurement reproducibility in ten 3DUS images. Each image was manually segmented using three different ISDs and repeatedly for two times at each of the three ISD settings: 1, 2, and 3 mm. In total, 60 MAB and LIB segmentation results (10 volumes × 2 repeat segmentations × 3 different ISDs) were obtained for the intra-observer variability analysis. The time interval between the repeated segmentations of the same volume was 2 weeks to minimize the effect of memory. Intra-observer reproducibility of repeated segmentations was evaluated in three ways, which are described in detail in the following three sub-sections: (a) reproducibility of manual carotid bifurcation localization, (b) segmentation reproducibility in terms of region-based and distance-based metrics, (c) consistency of VWV and VWT measurements computed from segmented boundaries.

#### 2.2.1. Reproducibility of Carotid Bifurcation Localization

The carotid bifurcation is where the CCA splits into the ICA and ECA. As manual segmentation was performed starting from the carotid bifurcation with a fixed ISD, the exact axial image slice segmented depended on the choice of the carotid bifurcation, which varies across trials of the same ISD. Therefore, it is important to study the reproducibility of carotid bifurcation localization across trials. In this study, the observer scanned through the 3DUS volume in the longitudinal direction to obtain a first approximation of the location of the bifurcation. After making this first approximation, the observer inspected the axial slice one ISD proximal to the approximate bifurcation and the axial slice one ISD distal to the approximate bifurcation. The observer adjusted the position of the bifurcation iteratively until there was a change of topology from the proximal slice to the distal slice. That is, only one artery (i.e., CCA) was observed in the proximal slice and two arteries (i.e., ICA and ECA) were observed to be completely separated in the distal slice. Typically, two to three iterations are required to determine the final location of the bifurcation. However, bifurcation localization became more difficult with multiple plaques near the carotid bifurcation, requiring the observer to go through more iterations to arrive at a bifurcation position. We quantified the variability in localizing the bifurcation by measuring the distance of the bifurcations identified in two segmentation trials of the same ISD along the longitudinal axis of the image.

#### 2.2.2. MAB and LIB Segmentation Reproducibility

**Figure 2:**
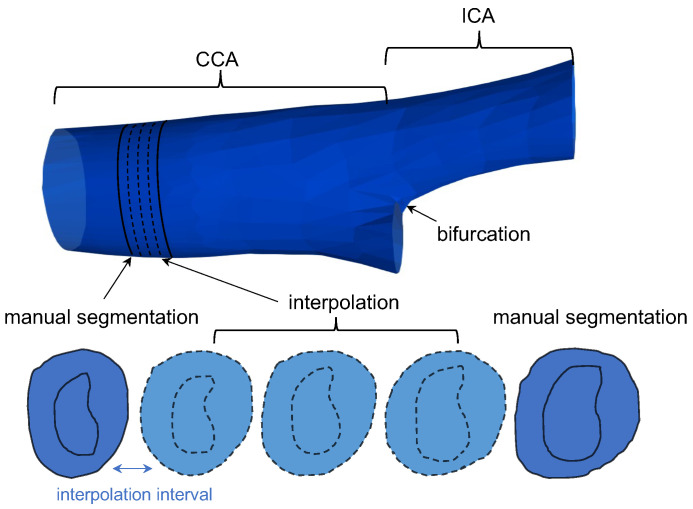
Shape-based interpolation applied to generate MAB and LIB between two neighboring manually segmented slices. The interpolation interval is equal to the voxel size in the longitudinal direction of the carotid volume.

The variability of two repeated segmentation trials of the same ISD was compared on a slice-by-slice basis. As the manual segmentation begins at the carotid bifurcation and the selection of the bifurcation varies across trials, the positions of the axial slices being segmented differ. Since fair comparison requires segmentation to be compared on common axial slices, we interpolated manually segmented MAB and LIB between two adjacent axial images in a 3DUS volume using the shape-based interpolation [38], as demonstrated in Figure 2. The interpolation interval is set to be the voxel size in the longitudinal direction of the carotid volume, denoted by $V_{z}$ (i.e., the common axial slices are separated by an ISD of $V_{z}$). On each common axial slice, the MAB and LIB associated with the two repeated trials were compared using the Dice similarity coefficient (DSC) and Hausdorff distance (HD) as defined below.

DSC is a region-based metric used to evaluate the area overlap between two boundaries:

(1) $$\mathrm{DSC}=2\frac{|M_{1}\cap M_{2}|}{|M_{1}\left|+\right|M_{2}|},$$

where $M_{1}$ and $M_{2}$ denote the region surrounded by two repeated segmentation boundaries, respectively, and |·| denotes the area of the region enclosed by the boundary. HD is used as a distance-based metric to assess the similarity between two segmented boundaries:

(2) $$\mathrm{HD}=max(h\left(M_{1},M_{2}\right),h\left(M_{2},M_{1}\right))$$

(3) $$h\left(M_{1},M_{2}\right)=\underset{m_{1}\in M_{1}}{max}\left\{\underset{m_{2}\in M_{2}}{min}\left\{d(m_{1},m_{2})\right\}\right\}$$

where $m_{1}$ and $m_{2}$ are points on the contour $M_{1}$ and $M_{2}$, respectively, and $d(m_{1},m_{2})$ is the Euclidean distance between $m_{1}$ and $m_{2}$.

#### 2.2.3. Reproducibility on VWV and VWT Measurements

As VWV and VWT measurements are shown to be sensitive to treatment effects [16,22], reproducible VWV and VWT measurements are important for patient monitoring and assessment based on 3DUS. In this study, we investigated the variability of VWV and VWT measured based on two repeated segmentations with the same ISD setting. The MAB and LIB contours in a carotid volume were reconstructed to 3D MAB and LIB surfaces using a previously proposed surface reconstruction algorithm [39]. The VWV measurement was calculated by subtracting the volume enclosed by the LIB surface from that enclosed by the MAB surface. To compute VWT, points on the MAB and LIB contours of each slice were matched using a symmetric correspondence algorithm [39], and the distance between a pair of corresponding points on the MAB and LIB was considered to be the point-by-point VWT. All the VWT measurements of a carotid volume can be superimposed on the corresponding MAB surface to generate the 3D VWT map, which can be projected onto a 2D carotid atlas using the arc-length scaling technique to obtain the L-shaped 2D VWT map [22]. The correlation and agreement of VWV and VWT measurements in repeated segmentations were assessed by the inter-class coefficient (ICC).

### 2.3. Algorithm Segmentation

Based on the results of the manual segmentation analysis described in Section 2.2, an ISD striking a balance between segmentation efficiency and the reproducibility of VWT and VWV measurements would be chosen as the ISD for manual segmentation, and manually segmented boundaries generated at this ISD were used to train the automated segmentation framework. The segmentation framework is described in Section 2.3.1, and the strategy aiming to improve the accuracy of the boundaries segmented by the framework is introduced in Section 2.3.2.

#### 2.3.1. Segmentation Framework

**Figure 3:**
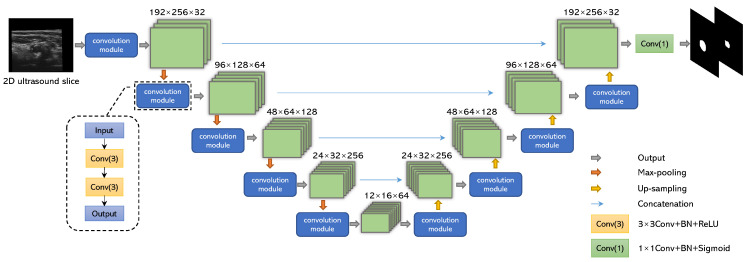
The architecture of the two-channel convolutional neural network. The number at the top of each block indicates the size of the tensor. Conv(n) represents a convolutional layer of size n×n, and BN represents batch normalization.

The automatic segmentation of the 3D carotid US images was performed with a two-channel U-Net, driven by the ATDL function proposed previously [33]. The structure of the network is shown in Figure 3. The input is the 2DUS slice, and the two-channel output represents the probabilities that each pixel is enclosed by the MAB and LIB, respectively. The two-channel network can segment LIB and MAB simultaneously, which is more efficient than using two independent models for MAB and LIB segmentations. A single network with two channels is more able to model the geometric relationship between the MAB and LIB (e.g., the LIB is always inside the MAB) than two independent networks segmenting the MAB and LIB separately [33]. The convolutional module consists of two convolutional layers with kernel size 3×3 and stride 1, each followed by a batch normalization (BN) layer and a ReLu activation layer. The down-sampling in the U-net utilizes 2D max-pooling of kernel size 2×2 with stride 2, and the up-sampling is conducted by 2D transposed convolution of kernel size 2×2 with stride 2. Due to the overlap between MAB and LIB, we use the sigmoid function instead of softmax as the activation function of the last layer because softmax requires the sum of the probabilities in all channels to be 1.

The ATDL used for supervising the training of the U-net has been described elsewhere [33], and briefly summarized here. ATDL is the adaptive Dice loss associated with the three components: MAB, LIB, and carotid vessel wall (CVW):

(4) $$L=aL_{\mathrm{DSC}}\left(y_{\mathrm{MAB}},{\hat{y}}_{\mathrm{MAB}}\right)+bL_{\mathrm{DSC}}\left(y_{\mathrm{LIB}},{\hat{y}}_{\mathrm{LIB}}\right)+cL_{\mathrm{DSC}}\left(y_{\mathrm{CVW}},{\hat{y}}_{\mathrm{CVW}}\right)$$

where $y_{\mathrm{MAB}}$, $y_{\mathrm{LIB}}$ and $y_{\mathrm{CVW}}$ are binary images that represent whether a pixel in the input image belongs to the region enclosed by manually segmented MAB, LIB, and CVW, respectively. CVW is the region between MAB and LIB, and $y_{\mathrm{CVM}}$ can be obtained by subtracting the $y_{\mathrm{LIB}}$ from the $y_{\mathrm{MAB}}$. ${\hat{y}}_{\mathrm{MAB}}$, ${\hat{y}}_{\mathrm{LIB}}$ and ${\hat{y}}_{\mathrm{CVW}}$ are the probabilities indicating the possibility that a pixel enclosed by MAB, LIB, and CVW, respectively. $L_{\mathrm{DSC}}\left(y,\hat{y}\right)$ is Dice loss defined by $L_{\mathrm{DSC}}\left(y,\hat{y}\right)=1-\frac{2y\times \hat{y}}{y+\hat{y}}$, where × and + represent pixel-wise multiplication and addition, respectively. *a*, *b*, and *c* are hyperparameters assigned to each Dice loss to balance the importance of the three Dice losses, and their sum equals 1. These hyperparameters were adaptively adjusted according to the loss value. The training process of ATDL supervision consists of two phases. At the beginning of training, uniform hyperparameters are used to facilitate the localization of MAB and LIB, since the value of $L_{\mathrm{DSC}}\left(y_{\mathrm{MAB}},{\hat{y}}_{\mathrm{MAB}}\right)$ and $L_{\mathrm{DSC}}\left(y_{\mathrm{LIB}},{\hat{y}}_{\mathrm{LIB}}\right)$ are larger than $L_{\mathrm{DSC}}\left(y_{\mathrm{CVW}},{\hat{y}}_{\mathrm{CVW}}\right)$ in the first phase. Later in the training process, $L_{\mathrm{DSC}}\left(y_{\mathrm{MAB}},{\hat{y}}_{\mathrm{MAB}}\right)$ and $L_{\mathrm{DSC}}\left(y_{\mathrm{LIB}},{\hat{y}}_{\mathrm{LIB}}\right)$ are rapidly reduced to values well below $L_{\mathrm{DSC}}\left(y_{\mathrm{CVW}},{\hat{y}}_{\mathrm{CVW}}\right)$. *a* and *b* are adjusted according to $L_{\mathrm{DSC}}\left(y_{\mathrm{MAB}},{\hat{y}}_{\mathrm{MAB}}\right)$ and $L_{\mathrm{DSC}}\left(y_{\mathrm{LIB}},{\hat{y}}_{\mathrm{LIB}}\right)$, respectively, using smaller values than *c*, so that the network can pay more attention to reducing $L_{\mathrm{DSC}}\left(y_{\mathrm{CVW}},{\hat{y}}_{\mathrm{CVW}}\right)$ in the second phase.

The data augmentation strategy is the same as that used in Jiang et al. [33]. First, the manually segmented MAB and LIB in the training set were reconstructed using a shape-based interpolation method [40], and the reconstructed surfaces were resliced with an ISD of 0.5 mm to generate additional segmented 2D images for training. Next, the geometric operations, including flipping, translation, and rotation, were performed to augment the training set further. Finally, horizontal and vertical flipping were used to augment the test set, resulting in three versions of the same image: the original, the horizontally, and vertically flipped images. The segmentation result generated for the flipped image can be flipped back to the original position so that there are three results to be selected for the same pixel point, and a majority vote is taken on a pixel basis to produce the final segmentation result for the 2D axial image.

The segmentation of CCA using the proposed U-net is fully automatic. However, the segmentation of the ICA requires two bounding boxes to be manually identified on the two ICA axial slices that are closest and furthest from the bifurcation. The rectangular boxes on the other ICA slices were automatically generated by linear interpolation. The manual interaction was required for ICA because it is difficult to distinguish the ICA and ECA on axial slices distal to the bifurcation. Since U-net requires the input images to have the same size, all input images were resampled to 192×256 pixels. The intensity of input images was normalized to [0, 1] by applying Min-Max normalization.

#### 2.3.2. Patient-Based vs. Time-Point-Based Partition

Serial monitoring of atherosclerosis requires 3DUS images to be acquired at baseline and follow-up scan sessions. In this study, we evaluated the hypothesis that training the network with the baseline images for segmentation of the follow-up images of the same cohort may provide higher segmentation accuracy than randomly splitting the training and test sets because the network could learn the basic geometry of the vessel from the baseline images corresponding to the test images. If the hypothesis is successfully validated, the “time-point-based” partitioning strategy would allow more accurate segmentation with an automatic framework, thereby generating more accurate VWV and VWT quantification for atherosclerosis monitoring.

**Figure 4:**
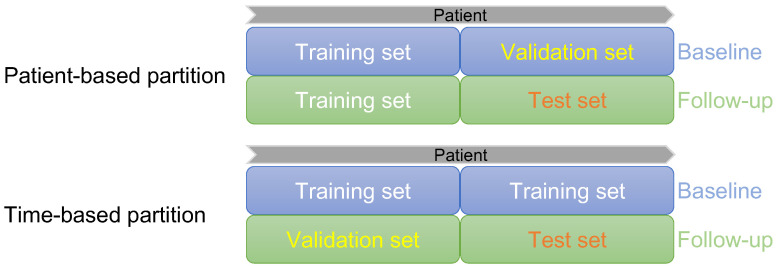
Illustration of two different data partitioning strategies.

We compared two different methods of dividing the dataset: the patient-based and the time-point-based partitions. Manually segmented boundaries of 84 3DUS volumes were used as the ground truth to train and evaluate the automatic segmentation algorithm. The 3DUS volumes obtained in baseline and follow-up were evenly divided into two parts for both partitioning strategies. As shown in Figure 4, patient-based partitioning involved using 1/2 of the baseline data and its corresponding follow-up data for training, and the other 1/2 of the baseline data and the follow-up data are used for the validation and testing, respectively. On the other hand, time-based partitioning involved using all the baseline data for training, while the follow-up data were partitioned into validation and testing sets. The same data are used for testing in both schemes for fair comparison.

### 2.4. Statistical Analysis

The Shapiro–Wilk test was used to test the normality of the DSCs of MAB and LIB segmentations obtained by comparing repeated segmentations. Paired *t*-tests were used for normally distributed continuous variables and the Wilcoxon signed ranks tests were used for non-normally distributed continuous variables to evaluate whether there were significant differences in the consistency of manual MAB and LIB segmentations across ISD settings. The difference was considered significant if the *p*-value was smaller than 0.05. In addition, the two-way mixed model intra-class correlation coefficient (ICC) with absolute agreement [41] was used to evaluate the agreement of VWV and VWT measurements obtained from manual segmentation. The value of ICC varies from 0 to 1, and excellent reliability is achieved with a value of 0.9 and higher, and good reliability is considered when the value is greater than or equal to 0.75 [41].

In evaluating algorithm segmentation, the DSC was computed by Equation (1), but different from intra-observer reproducibility assessment, $M_{1}$ and $M_{2}$ were, respectively, set to be the algorithm and manually segmented contours. The DSCs attained by the time-based and patient-based partitioning scheme were statistically compared either by the paired *t*-test or the Wilcoxon signed ranks test depending on the normality of the data set. The difference was considered significant if the probability of making a type I error was less than 0.05.

The Pearson correlation coefficient (*r*) and Bland–Altman analysis were used to assess the agreement between VWT and VWV measured from manual and algorithm segmentations. The *p*-value associated with *r* was used to show the significance of the correlation. The Bland–Altman plots were generated using the difference and average of the two sets of VWV/VWT measurements. The repeatability coefficient (RPC) and coefficient of variation (CV) were computed from the Bland–Altman plot [42]. RPC is defined as 1.96 times the standard deviation (SD) of the difference between the measurements, and CV is measured as the SD of the difference divided by the mean of the two sets of measurements, expressed as a percentage. We also evaluated the bias and the accuracy of VWV and VWT measured from algorithm segmentation by the difference (denoted by ΔVWV and ΔVWT) and absolute difference (denoted by |ΔVWV| and |ΔVWT|), respectively, between the measurements obtained from the algorithm and manual segmentations.

## 3. Results

### 3.1. Manual Segmentation Evaluation

**Figure 5:**
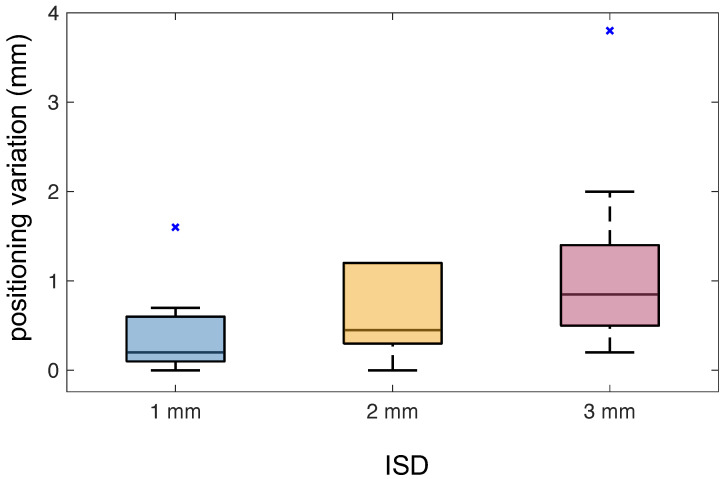
The differences in bifurcation positioning at three different ISD settings.

Figure 5 shows the differences in the bifurcation location of ten volumes in two repeated segmentations under different ISD settings. The mean distance of the bifurcations identified in the two trials increased with the ISD. The mean distances of the bifurcation location were 0.40 mm, 0.62 mm, and 1.19 mm for ISD setting to 1 mm, 2 mm, and 3 mm, respectively.

Table 1 shows the DSC and HD of two repeated segmentations of MAB and LIB for each of the three different ISD settings. MAB is more reproducible than LIB, and both MAB and LIB are more reproducible in CCA than in ICA. Table 2 shows the *p*-values of the paired *t*-tests performed on DSCs of MAB and LIB segmentations for three pairs of ISD settings. Paired *t*-tests were used for all comparisons as all DSCs were found to be normal. No significant difference in manual segmentation consistency was found between the ISD settings of 1 mm and 2 mm, while significant differences were detected between the ISDs of 1 mm and 3 mm, and between the ISDs of 2 mm and 3 mm.

We measured VWV from the segmentation results of ten ultrasound volumes. The intra-observer ICC calculated using these measurements is shown in Table 3. The ICC values of the observer for the VWV measurement at different ISDs are all above 0.9, but the decrease was substantial from 2 mm to 3 mm. The width of the confidence interval (CI) has also increased substantially.

**Figure 6:**
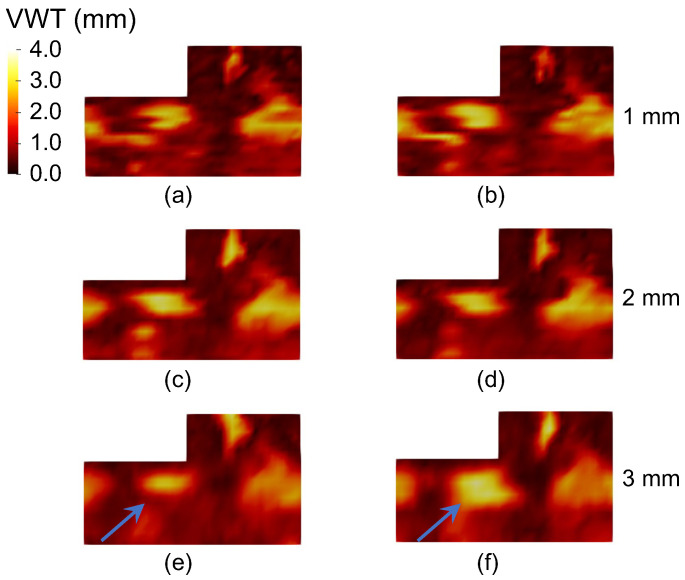
The VWT maps generated for an example 3DUS volume based on the manual segmentation results at (**a**,**b**) ISD = 1 mm, (**c**,**d**) 2 mm, and (**e**,**f**) 3 mm. Segmentation was performed twice for each ISD setting. Comparison of (**e**,**f**) shows a plaque associated with large segmentation variability in two repeated segmentation trials at ISD = 3 mm.

Table 4 shows the intra-observer of VWT measurements and Figure 6 shows the 2D VWT maps generated for an example 3DUS volume from the six manual segmentations (i.e., 2 repeated segmentations × 3 ISD settings). The ICC values for VWT were all lower than VWV as each VWT map was equipped with several hundred local measurements, thereby showing dense variation not captured by the more global VWV measurement. The ICC for VWT at 2 mm ISD was higher than that obtained at 1 mm. This may stem from the fact that the VWT map obtained with the 1 mm ISD contains denser point-wise VWT measurements, thereby showing more detailed variation than the VWT map obtained with the 2 mm ISD, as shown in Figure 6a–d. Table 4 shows that the ICC of VWT decreases substantially at ISD = 3 mm. An ISD of 3 mm is comparable to the longitudinal length of some small plaques. The area occupied by such a plaque in the VWT map varies depending on whether the plaque is delineated on one or two adjacent axial slices. Figure 6e,f shows a plaque segmented at two repeated segmentation trials. The plaque is pointed to by the blue arrows. Figure 6e shows a small plaque delineated at only one axial slice, whereas the plaque was segmented at two adjacent axial slices in the second segmentation trial at ISD = 3 mm for the same patient shown in Figure 6f.

### 3.2. Algorithm Segmentation Evaluation

Based on the reproducibility of the manual segmentation reported in Section 3.1, we concluded that a balance between segmentation efficiency and reproducibility is best achieved at ISD = 2 mm. Thus, we evaluated the segmentation performance of deep neural networks using manual boundaries segmented at 2 mm ISD. Table 5 shows the DSC and HD for algorithm segmentation using different data partitioning strategies. BF in Table 5 stands for the bifurcation slice and is defined as the CCA slice most proximal to the bifurcation, where the CCA is about to separate into the ICA and ECA. Both metrics indicate that better accuracy can be obtained by the time-based splitting strategy, with considerable improvements for CCA segmentation, especially in the bifurcation slices. Table 6 shows the *p*-values of paired-sample tests performed to compare results of the subject- and time-based partitions. There were significant improvements in the accuracy of the MAB and LIB segmentations in CCA and LIB segmentation in ICA by the time-based partition over the patient-based partition. However, the improvement in the MAB segmentation at ICA was not statistically significant. Since an ROI was manually identified to enclose the ICA, the accuracy of the MAB at ICA segmented by the patient-based partition strategy already approached that of manual segmentation (Manual: 92.91%±3.30%; Patient-based partition: 92.27%±3.79%), and the time-based partitioning strategy could not further improve the MAB segmentation of ICA.

**Figure 7:**
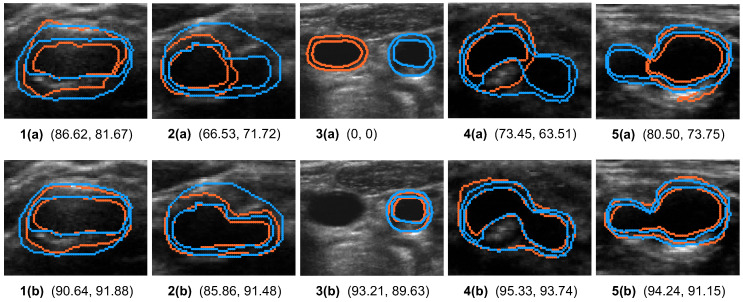
The effect of the time-based partitioning strategy on the performance of CCA segmentation is demonstrated in five example images. Images shown in the same column corresponds to the same example with segmentation results generated by (**a**) the patient-based and (**b**) the time-based partitioning strategies. The blue and orange contours represent manual and automatic segmentations, respectively. Numbers below each image represent DSC for MAB and LIB segmentations in percentage.

The improvement in the BF slice was more pronounced than the non-BF CCA slices, as shown in Table 5, but the *p*-value for non-BF CCA slices is smaller than that for the BF slices in Table 6 due to the sample size difference. There were more than 10 CCA slices per volume, whereas there was only one BF slice per volume. The boundaries segmented by the two data splitting strategies were compared quantitatively in five examples shown in Figure 7. The first two examples are BF slices, and the remaining three are non-BF CCA slices. The MAB and LIB on the BF slices segmented by the patient-based data partitioning strategy missed large sections of the carotid artery, and the segmentation inaccuracies were largely corrected by the time-based partitioning strategy. In the presence of other vessels in Figure 7b, the CCA was correctly localized by the time-based partitioning strategy, but not the patient-based partitioning strategy.

**Figure 8:**
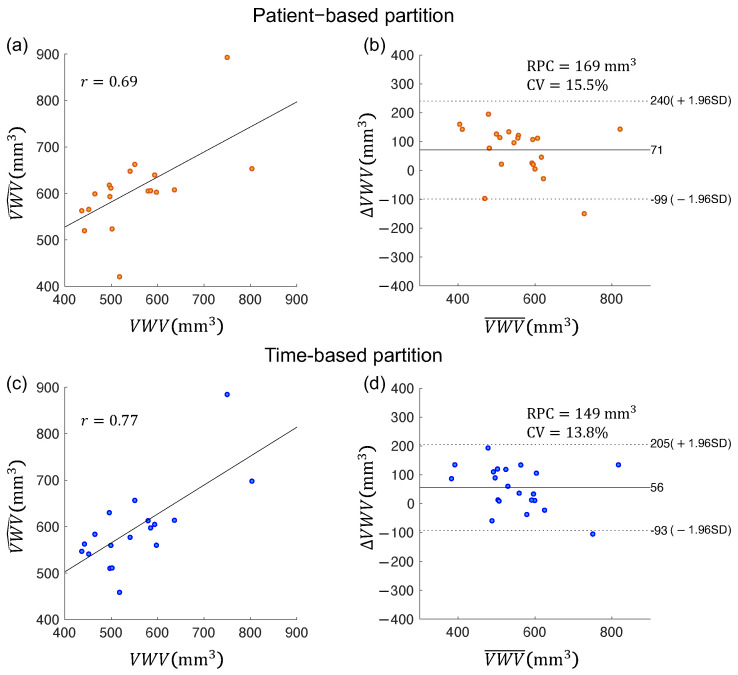
(**a**,**c**) Pearson’s correlation plots and (**b**,**d**) Bland–Altman plots for vessel wall volume measurements obtained from manual segmentation (VWV) and those obtained from two algorithm segmentation methods ($\hat{VWV}$), with algorithm segmentation performed using (**a**,**b**) patient-based and (**c**,**d**) time-based partitioning strategies, respectively.

**Figure 9:**
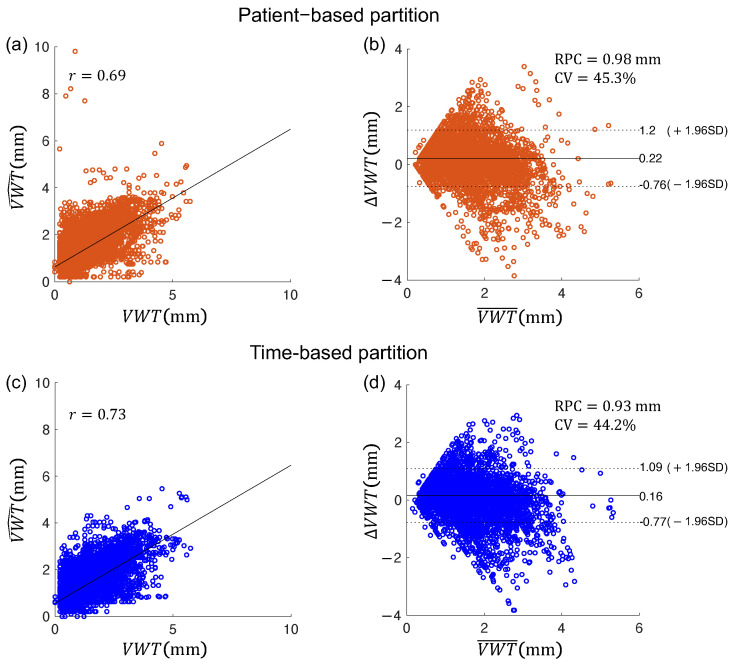
(**a**,**c**) Pearson’s correlation plots and (**b**,**d**) Bland–Altman plots for vessel-wall-plus-plaque thickness measurements obtained from manual segmentation (VWT) and those obtained from two algorithm segmentation methods ($\hat{VWT}$), with algorithm segmentation performed using (**a**,**b**) patient-based and (**c**,**d**) time-based partitioning strategies, respectively.

The VWV and VWT measured from the contours segmented using two data partitioning strategies were compared with the corresponding measurements from manual segmentation. Figure 8 shows the correlation and Bland–Altman plots for VWV measurements generated from manually segmented boundaries (denoted by VWV) and those generated from algorithm segmented boundaries (denoted by $\hat{VWV}$). $\Delta VWV=\hat{VWV}-VWV$ and $\bar{VWV}=1/2\left(\hat{VWV}+VWV\right)$ denote the difference and average of the two VWV measurements, respectively. Figure 9 shows similar plots for the VWT measurements. The VWT map of a carotid volume contains hundreds of localized measurements, whereas a volume has only a single VWV measurement. The correlations of the measurements obtained by the time-based partitioning strategy with manual measurements shown in Figure 8c, Figure 9c and Table 7 were higher than those obtained by the patient-based partitioning strategy in Figure 8a, Figure 9a and Table 7. The Bland–Altman plots show the time-based partitioning strategy in Figure 8d and Figure 9d produced smaller bias and ranges of agreement for VWV and VWT measurements than the patient-based segmentation strategy in Figure 8b and Figure 9b. The time-based partitioning strategy also yielded smaller RPC and CV values for both VWV and VWT measurements, indicating better agreement with the manual measurements compared to the patient-based partitioning strategy. Table 7 lists the differences and correlations of the VWV and VWT measured from the algorithm segmentation and from manual segmentation. The VWV and VWT measurements obtained by the time-based partitioning strategy were associated with smaller bias and absolute error. The correlations of the measurements obtained by the time-based partitioning strategy with manual measurements were also higher than those obtained by the patient-based partitioning strategy.

## 4. Discussion

VWV and VWT are quantitative 3DUS measurements useful for monitoring changes of carotid atherosclerosis and they were shown to be sensitive in detecting the effects of medical therapies and dietary interventions [16,22,43]. A major bottleneck in previous clinical studies involving 3D carotid US assessment is the requirement to segment the MAB and LIB manually. While CNN segmentation networks have been developed to provide automatic segmentation, they still require supervision by manually segmented boundaries. Therefore, there is a critical requirement to design a 3DUS carotid image analysis workflow to improve the efficiency of generating manual segmentation. While segmentation time can be reduced by increasing the interval at which axial slices are segmented (i.e., increasing ISD), segmentation reliability may be compromised as adjacent axial slices with a larger ISD may appear less continuous and more difficult to segment. The first question addressed in this study is to what extent we could increase the ISD (therefore reducing segmentation time) while maintaining an acceptable level of the reliability of the boundaries used to train the network. The current study assessed the effect of ISD on the intra-observer reproducibility of MAB and LIB segmentations and the VWV and VWT measured from the segmented MAB and LIB. We established that the intra-observer reproducibility of MAB and LIB segmentations, quantified by DSC, at ISDs of 1 mm and 2 mm was not statistically significantly different, whereas the reproducibilities at ISD = 1 mm and 2 mm were both statistically significantly higher than that attained at ISD = 3 mm. The ICCs of VWV and VWT measurements obtained at an ISD of 3 mm were also substantially lower than those attained at the ISD of 1 mm and 2 mm. Our result suggests that manual segmentation reliability at ISD = 2 mm is similar to that at ISD = 1 mm, and the reliability substantially reduces with the ISD increasing to 3 mm. As setting the ISD at 2 mm saves half of the segmentation time compared to 1 mm and we attempt to optimize for efficiency of the automated 3DUS analysis workflow, we applied an ISD of 2 mm for generating training boundaries when evaluating the CNN segmentation network.

Random partitioning of the available data into training and testing sets allows a machine learning model to generalize to new patient data. Furthermore, for a fair evaluation of the machine learning model, different data items associated with a patient are partitioned into the same set. However, the considerations involved in designing a segmentation model suitable for *serial* monitoring of patients are slightly different from a typical machine learning model. Instead of being able to generalize to new patient data, a more important consideration is higher segmentation accuracy in a batch of data acquired at a follow-up time point for the same cohort of patients involved in training. For this reason, we proposed training our segmentation network with the baseline images of the entire cohort of patients and testing the network with the follow-up images of the same cohort of patients. In such a partitioning scheme we call *time-based* partitioning, the network is exposed to the geometry of the carotid vessel in the entire cohort of patients, and therefore, is expected to perform better in segmenting the follow-up images for the same cohort of patients. We compared time-based partitioning with the more conventional patient-based partitioning in evaluating our segmentation model. Our result shows that the segmentation model under the time-based partitioning scheme was better at segmenting more complex structures, such as the carotid bifurcation, than under the patient-based partitioning scheme. As the artery is about to divide from the CCA into the ICA and ECA at the bifurcation, the carotid geometry is highly elongated at the carotid bifurcation; additionally, as plaques are prone to develop at the bifurcation, the shape of the LIB here can be highly irregular (e.g., Figure 7a). As the bifurcation is a key location to be characterized in carotid atherosclerosis monitoring and assessment, higher segmentation performance in this location by the time-based partitioning scheme suggests that a better workflow for carotid analysis is to train the network using baseline images of the entire cohort and use the trained network for automatic segmentation for follow-up images of the same cohort. This workflow would be particularly cost-effective if serial monitoring is carried out at multiple follow-up time points.

**Figure 10:**
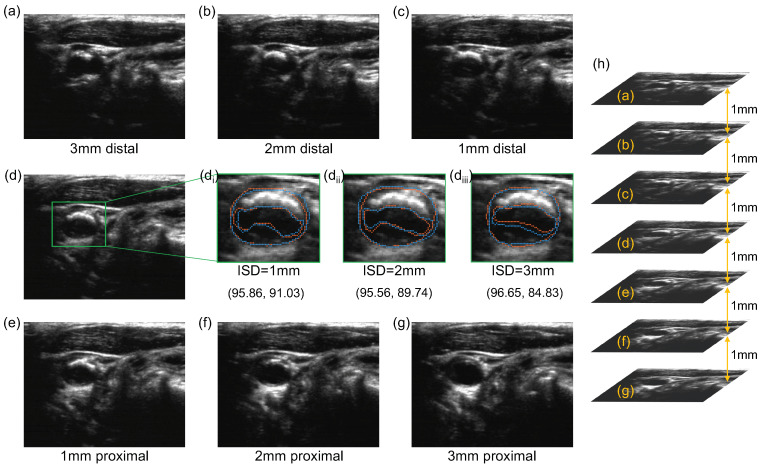
MAB and LIB segmentation reproducibility in an example image labeled by (**d**) with a calcified plaque on the near wall and a hypo-echoic plaque on the far wall. Figures ($d_{i}$–$d_{\mathrm{iii}}$) show the two repeated MAB and LIB manual segmentations obtained at ISD = 1, 2 and 3 mm, respectively, each with the DSCs of the MAB and LIB provided in the bracket underneath the corresponding image. The first and second numbers in the brackets correspond to the DSC for MAB and LIB segmentations, respectively. The distal images associated with the ISD of 3, 2 and 1 mm are shown in (**a**–**c**), respectively, whereas the proximal images associated with the ISD of 1, 2 and 3 mm are shown in (**e**–**g**), respectively. Figure (**h**) shows the relative positions of the resliced axial images shown in (**a**–**g**).

We reported that the DSCs of manual MAB and LIB segmentations at ISD = 1 and 2 mm were statistically different from the DSCs at ISD = 3 mm. Additionally, the standard deviations of DSC and HD of LIB segmentation at 3 mm ISD setting were substantially larger than at 1 and 2 mm, as shown in Table 1. There are two possible explanations for these observations. The first is related to the presence of calcified and hypo-echoic plaques, as demonstrated in Figure 10. The presence of calcified plaques leads to acoustic shadowing. The use of compounding imaging mitigated the effect of acoustic shadowing [44] and in this study, acoustic shadowing mostly appears beyond the arteries and does not substantially obscure the wall and lumen. In acquiring 3DUS images, the sonographer identifies plaques based on Doppler images and attempts to adjust the contrast and level of the ultrasound images before an observer or an algorithm segments boundaries. This adjustment mitigates the difficulty in segmenting hypo-echoic plaques. Despite the use of these strategies, the presence of these two types of plaques is expected to increase observer variability. The availability of 3DUS images allows observers to review adjacent slices, thereby providing visual clues that better equip them to trace out obscured boundaries more reproducibly, as demonstrated in the segmentation of the image shown in Figure 10d that has a calcified plaque in the near wall and a hypo-echoic plaque in the far wall. However, the observer variability in segmenting the hypo-echoic plaque was substantially larger at ISD = 3 mm than at ISD = 1 and 2 mm. The distal images associated with the ISD of 3, 2 and 1 mm are shown in Figure 10a–c, respectively, whereas the proximal images associated with the ISD of 1, 2 and 3 mm are shown in Figure 10e–g, respectively. At ISD = 3 mm, the distal (Figure 10a) and the proximal (Figure 10g) provided limited visual clues for LIB segmentation, thereby leading to a decrease in the DSC of the repeated LIB segmentations by approximately 5% compared to when the ISD is set to 1 or 2 mm. The second explanation of the increased variability at ISD = 3 mm is related to the existence of plaques with small longitudinal lengths as shown in Figure 6. With ISD = 3 mm, there were either one or two resliced planes intersecting these plaques. The variability in the number of planes intersecting them in different segmentation sessions contributed to a large variability in their longitudinal coverage, which in turn reduced the reproducibility in LIB segmentation.

Related to the reflection of the ultrasound beam by calcified plaques discussed above, the literature has described that highly reflective structures, such as calcification, may expand beyond their true boundaries and appear larger in an image [45]. This effect, commonly referred to as the blooming effect, is less of an issue in our study. The image intensity adjustment based on Doppler imaging by the sonographer ensures the size of a calcified plaque is appropriately displayed in the ultrasound image. The image intensity adjustment also benefited from compounding imaging, which combines images scanned at different angles. Compounding imaging has been shown to improve the definition of plaque surfaces [44]. As demonstrated in Figure 10, the observer segmenting MAB and LIB was also helped by the ability to observe adjacent axial images in a 3DUS image.

In this study, we applied the segmentation model we previously developed [33] to evaluate the proposed time-based partitioning approach. The DSC attained in the current study are 92.3%±5.7% and 89.1%±6.4% for the MAB and LIB in CCA segmentation and 92.6%±3.6% and 89.5%±3.8% for the MAB and LIB in ICA segmentation. The results are similar to those attained for a cohort studied in Jiang et al. [33] with asymptomatic carotid stenosis involved in a placebo-controlled trial evaluating the effect of atorvastatin (CCA: 92.3%±9.8% for MAB and 88.1%±13.7% for LIB; ICA: 93.2%±2.9% for MAB and 87.3%±12.1% for LIB). The quantification of the intra-observer variability in MAB and LIB segmentations in the current study (Table 1) affords us an opportunity to assess the performance of the segmentation network in relation to observer variability. The DSC attainable by the segmentation framework is close to the DSC attributable to intra-observer variability for ICA segmentation, whereas the DSC of the segmentation framework for CCA segmentation is 2–3% lower than the DSC associated with intra-observer variability. The ICA was enclosed by a manually identified ROI before the application of the segmentation network, and therefore, localization of the ICA was less of an issue for the segmentation network. On the other hand, the segmentation network is not provided an ROI for CCA segmentation and there is a chance that the CCA is mislocalized as demonstrated in Figure 7. Figure 7 also shows that the artery at the bifurcation typically has a more complex shape and the network sometimes fails to segment the entire artery. These two factors contributed to the reduced DSC attained by the segmentation network as compared to the DSC associated with intra-observer variability.

When segmenting a given axial image, the observer has knowledge of the arterial shape on adjacent axial slices. The segmentation network evaluated in this study, however, is a 2D segmentation network processing each slice independently. We applied the 2D network in this study because the major focus of this paper is to evaluate how training and inference should be made to improve segmentation accuracy in series assessment of 3D carotid US images. Further improvement to the segmentation performance may be provided by segmentation networks that consider neighboring slices as well as the current axial slice being segmentation (2.5D network) or 3D segmentation network [46] and improvement of the time-based partitioning approach in these networks are required to be evaluated in a future study. We did not include an inter-observer reproducibility analysis when assessing the effect of different ISDs on segmentation reproducibility. Chan et al. [47] performed an extensive study to evaluate the inter-observer reproducibility in measuring VWV from 3DUS and found that the coefficients of variance of VWV measured by five observers were similar. In the current study, the focus was more on assessing the impact of ISD on manual segmentation reliability; with the results from Chan’s study indicating the similarity of VWV reproducibility measured by multiple observers under the same protocol, we decided not to perform an intra-observer analysis, as manual segmentation by multiple observers would require long training and implementation times. Additionally, a central reading center involving a standard grading protocol is typically used for multi-center clinical trials involving imaging endpoints [48].

## 5. Conclusions

We, for the first time, investigated the effect of ISD between segmented axial images on the reproducibility of the segmented boundaries and associated VWV and VWT measurements. We determined that an ISD of 2 mm provides sufficient reliability of the boundaries for CNN training, reducing the segmentation time by half compared to previous clinical studies with an ISD of 1 mm [14,16,18,47]. We further proposed a workflow to train the CNN by the baseline images of the entire cohort for automatic segmentation of the follow-up images of the same cohort and showed improved accuracy of this *time-based* training approach as compared to the more conventional *patient-based* approach. The proposed analysis workflow was tailored for *serial* monitoring of carotid atherosclerosis and can be useful in clinical studies evaluating the effect of new treatments, such as [22,23,47] and risk stratification studies, such as [35].

## Figures and Tables

**Table 1:** The means and the standard deviations of Dice similarity coefficient (DSC) and Hausdorff distance (HD) in repeated MAB and LIB segmentations at three different ISD settings.

ISD		DSC (%)		HD (mm)	
		**MAB**	**LIB**	**MAB**	**LIB**
CCA	1 mm	95.45 (1.66)	92.23 (3.39)	0.68 (0.26)	0.85 (0.42)
	2 mm	95.04 (1.59)	91.90 (3.82)	0.72 (0.26)	0.85 (0.41)
	3 mm	95.03 (2.04)	90.32 (4.41)	0.73 (0.30)	0.98 (0.44)
ICA	1 mm	92.51 (3.46)	89.95 (5.49)	0.67 (0.25)	0.69 (0.32)
	2 mm	92.91 (3.30)	91.60 (4.54)	0.59 (0.22)	0.63 (0.43)
	3 mm	91.72 (4.25)	88.19 (11.69)	0.74 (0.38)	0.78 (0.65)
ALL	1 mm	94.63 (2.65)	91.59 (4.20)	0.67 (0.26)	0.80 (0.40)
	2 mm	94.45 (2.40)	91.82 (4.02)	0.68 (0.24)	0.79 (0.42)
	3 mm	94.11 (3.19)	89.73 (7.26)	0.73 (0.32)	0.93 (0.52)

**Table 2:** The *p*-values of the paired *t*-tests performed on the Dice similarity coefficient (DSC) for manual MAB and LIB segmentations under 3 pairs of ISD settings.

ISD		DSC *p*-Value	
		**MAB**	**LIB**
ALL	1 mm & 2 mm	0.140	0.203
	1 mm & 3 mm	0.001	3.498 × 10 ${}^{-9}$
	2 mm & 3 mm	0.008	2.898 ×10 ${}^{-9}$

**Table 3:** Intra-observer ICC values for VWV measurements at different ISD settings.

ISD	Intraclass Correlation Coefficient	95% Confidence Interval	
		**Lower Bound**	**Upper Bound**
1 mm	0.979	0.922	0.995
2 mm	0.961	0.861	0.990
3 mm	0.911	0.688	0.977

**Table 4:** Intra-observer ICC values for VWT measurements at different ISD settings.

ISD	Intraclass Correlation Coefficient	95% Confidence Interval	
		**Lower Bound**	**Upper Bound**
1 mm	0.850	0.844	0.856
2 mm	0.887	0.882	0.891
3 mm	0.837	0.831	0.844

**Table 5:** The means and the standard deviations of Dice coefficient (DSC) and Hausdorff distance (HD) in algorithm MAB and LIB segmentations using two different data partitioning strategies. BF is the collection of the first CCA slices most proximal to the bifurcation. CCA w/o BF excludes the BF slices and only considers non-BF CCA slices.

Partitioning Scheme		DSC (%)		HD (mm)	
		**MAB**	**LIB**	**MAB**	**LIB**
Patient-based partition	CCA	89.92 (10.60)	86.27 (11.61)	1.66 (2.06)	1.70 (2.07)
	ICA	92.27 (3.79)	88.66 (4.61)	0.80 (0.34)	0.89 (0.42)
	BF	83.02 (10.10)	80.32 (12.19)	3.41 (2.71)	3.28 (2.96)
	CCA w/o BF	90.91 (10.33)	87.12 (11.31)	1.41 (1.83)	1.48 (1.81)
	Overall	90.83 (8.70)	87.19 (9.60)	1.33 (1.68)	1.39 (1.69)
Time-based partition	CCA	92.34 (5.69)	89.10 (6.43)	1.23 (1.31)	1.29 (1.27)
	ICA	92.59 (3.61)	89.54 (3.77)	0.77 (0.30)	0.82 (0.33)
	BF	89.35 (6.98)	87.35 (6.61)	1.94 (1.95)	1.82 (2.13)
	CCA w/o BF	92.77 (5.37)	89.36 (6.39)	1.13 (1.16)	1.21 (1.09)
	Overall	92.44 (4.98)	89.27 (5.56)	1.06 (1.05)	1.11 (1.06)

**Table 6:** The *p*-values of Dice coefficient (DSC) for algorithm MAB and LIB segmentations between time- and patient-based partitioning. CCA w/o BF excludes the BF slices and only considers non-BF CCA slices.

	DSC *p*-Value	
	MAB	LIB
CCA	$1.27\times 10^{-9}$	$1.74\times 10^{-7}$
ICA	0.08	$4.45\times 10^{-4}$
BF	0.004	0.04
CCA w/o BF	$7.95\times 10^{-8}$	$8.59\times 10^{-7}$
ALL	$1.16\times 10^{-9}$	$6.22\times 10^{-10}$

**Table 7:** The means and the standard deviations of VWV and VWT difference (in mm${}^{3}$ and mm, respectively) between two algorithm segmentation strategies (i.e., time-based and patient-based partitioning) and manual segmentation. *r* is the Pearson’s correlation coefficient, and the associated *p*-value shows the significance of the correlation.

Partitioning Scheme	ΔVWV	|ΔVWV|	*r*	*p*-Value
Patient-based partition	70.71 (86.42)	96.97 (53.35)	0.69	$5.56\times 10^{-4}$
Time-based partition	56.13 (76.12)	77.52 (52.84)	0.77	<1.0 $\times 10^{-4}$
**Partitioning Scheme**	**ΔVWT**	**|ΔVWT|**	***r***	***p*** **-Value**
Patient-based partition	0.22 (0.50)	0.39 (0.38)	0.69	<1.0 $\times 10^{-4}$
Time-based partition	0.16 (0.47)	0.36 (0.35)	0.73	<1.0 $\times 10^{-4}$

## Data Availability

The data that support the findings of this study are available from the Robarts Research Institute, London, Ontario, Canada. Restrictions apply to the availability of these data, which were used by permission for this study. Data are available from the corresponding author on reasonable request and with the permission of the Research Ethics Board of Western University.
